# Supplementary material for: Time-series transcriptome analysis identified differentially expressed genes in broiler chicken infected with mixed Eimeria species
Source: Front Genet. 2022 Aug 8;13:886781. doi: 10.3389/fgene.2022.886781 (PMC9393255; doi:10.3389/fgene.2022.886781)
Supplement: Supplementary file 2 [file DataSheet1.ZIP › 7dpi_KEGG.Gsea.1625115961950/KEGG_AMINO_SUGAR_AND_NUCLEOTIDE_SUGAR_METABOLISM.html]

Details for gene set KEGG\_AMINO\_SUGAR\_AND\_NUCLEOTIDE\_SUGAR\_METABOLISM[GSEA]

|  || Dataset | TMM\_7dpi\_gct\_format\_7dpi\_gct\_format.Class\_4dpi.cls #PC\_versus\_NC.Class\_4dpi.cls #PC\_versus\_NC\_repos |
| Phenotype | Class\_4dpi.cls#PC\_versus\_NC\_repos |
| Upregulated in class | 1 |
| GeneSet | KEGG\_AMINO\_SUGAR\_AND\_NUCLEOTIDE\_SUGAR\_METABOLISM |
| Enrichment Score (ES) | 0.5100681 |
| Normalized Enrichment Score (NES) | 2.1444178 |
| Nominal p-value | 0.0 |
| FDR q-value | 0.0012285105 |
| FWER p-Value | 0.0066 |
Table: GSEA Results Summary

  

Fig 1: Enrichment plot: KEGG\_AMINO\_SUGAR\_AND\_NUCLEOTIDE\_SUGAR\_METABOLISM      
 Profile of the Running ES Score & Positions of GeneSet Members on the Rank Ordered List

  

| SYMBOL | TITLE | RANK IN GENE LIST | RANK METRIC SCORE | RUNNING ES | CORE ENRICHMENT || 1 | NPL | na | 44 | 1.481 | 0.0669 | Yes |
| 2 | GMDS | na | 105 | 1.237 | 0.1208 | Yes |
| 3 | UGDH | na | 117 | 1.206 | 0.1773 | Yes |
| 4 | CHIA | na | 130 | 1.172 | 0.2321 | Yes |
| 5 | NANS | na | 135 | 1.164 | 0.2872 | Yes |
| 6 | GCK | na | 150 | 1.139 | 0.3403 | Yes |
| 7 | UAP1 | na | 278 | 0.957 | 0.3753 | Yes |
| 8 | HK1 | na | 361 | 0.870 | 0.4099 | Yes |
| 9 | GFPT2 | na | 567 | 0.700 | 0.4261 | Yes |
| 10 | PGM3 | na | 583 | 0.693 | 0.4578 | Yes |
| 11 | MPI | na | 685 | 0.642 | 0.4800 | Yes |
| 12 | GALK1 | na | 691 | 0.640 | 0.5101 | Yes |
| 13 | GALE | na | 1315 | 0.426 | 0.4783 | No |
| 14 | GMPPB | na | 2196 | 0.237 | 0.4161 | No |
| 15 | GNPNAT1 | na | 2805 | 0.150 | 0.3725 | No |
| 16 | CYB5R1 | na | 2830 | 0.147 | 0.3775 | No |
| 17 | PMM2 | na | 3285 | 0.090 | 0.3438 | No |
| 18 | GMPPA | na | 3585 | 0.054 | 0.3214 | No |
| 19 | CMAS | na | 3614 | 0.051 | 0.3215 | No |
| 20 | GALK2 | na | 3951 | 0.010 | 0.2939 | No |
| 21 | HK3 | na | 6191 | -0.224 | 0.1176 | No |
| 22 | UXS1 | na | 6259 | -0.230 | 0.1230 | No |
| 23 | NANP | na | 6578 | -0.262 | 0.1089 | No |
| 24 | GPI | na | 6921 | -0.299 | 0.0946 | No |
| 25 | UGP2 | na | 7288 | -0.339 | 0.0802 | No |
| 26 | GALT | na | 7330 | -0.344 | 0.0932 | No |
| 27 | GNPDA2 | na | 7623 | -0.374 | 0.0866 | No |
| 28 | PGM1 | na | 7809 | -0.397 | 0.0900 | No |
| 29 | FPGT | na | 7879 | -0.405 | 0.1036 | No |
| 30 | AMDHD2 | na | 7913 | -0.410 | 0.1203 | No |
| 31 | HK2 | na | 8169 | -0.442 | 0.1201 | No |
| 32 | GNPDA1 | na | 8351 | -0.465 | 0.1272 | No |
| 33 | GFPT1 | na | 8396 | -0.471 | 0.1459 | No |
| 34 | GNE | na | 8603 | -0.500 | 0.1525 | No |
| 35 | HEXB | na | 8834 | -0.528 | 0.1585 | No |
| 36 | PMM1 | na | 9046 | -0.556 | 0.1673 | No |
| 37 | HEXA | na | 10495 | -0.808 | 0.0849 | No |
| 38 | PGM2 | na | 10778 | -0.873 | 0.1029 | No |
Table: GSEA details [plain text format]

  

Fig 2: KEGG\_AMINO\_SUGAR\_AND\_NUCLEOTIDE\_SUGAR\_METABOLISM      
 Blue-Pink O' Gram in the Space of the Analyzed GeneSet

  

Fig 3: KEGG\_AMINO\_SUGAR\_AND\_NUCLEOTIDE\_SUGAR\_METABOLISM: Random ES distribution      
 Gene set null distribution of ES for **KEGG\_AMINO\_SUGAR\_AND\_NUCLEOTIDE\_SUGAR\_METABOLISM**
